# Supplementary material for: Simulation and empirical evaluation of biologically-informed neural network performance
Source: bioRxiv. 2025 Nov 14:2025.11.13.687845. Preprint. [Version 1] doi: 10.1101/2025.11.13.687845 (PMC12642320; doi:10.1101/2025.11.13.687845)
Supplement: 7 [file NIHPP2025.11.13.687845v1-supplement-7.pdf]

bioRxiv preprint doi: https://doi.org/10.1101/2025.11.13.687845; this version posted November 14, 2025. The copyright holder for this preprint (which was not certified by peer review) is the author/funder, who has granted bioRxiv a license to display the preprint in perpetuity. It is made available under aCC-BY 4.0 International license.

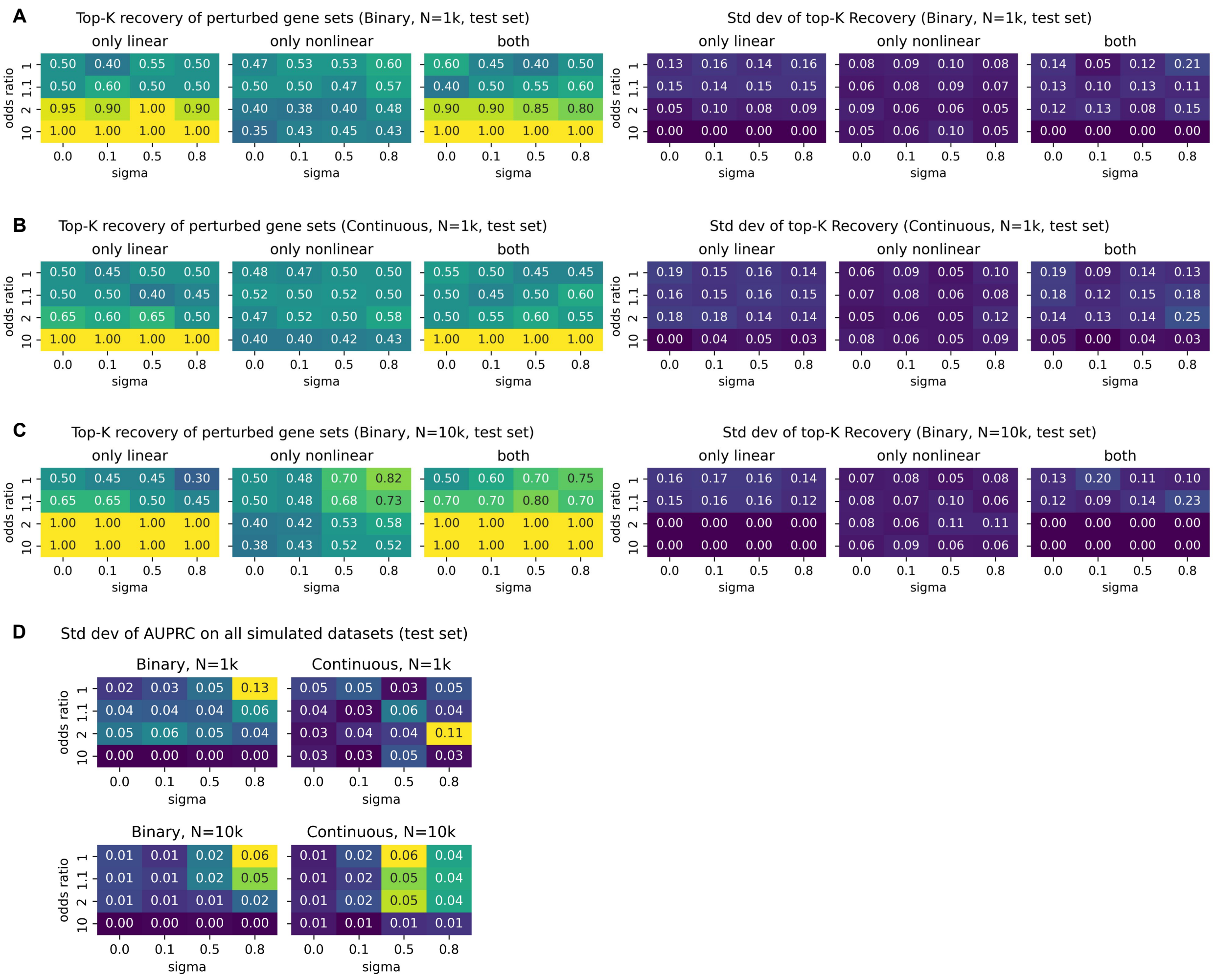

Figure S1. Top-K recovery of P-NET in simulated datasets with varied signal type and strength. Each subfigure shows the top-K recovery of three ground-truth gene sets across all tested combinations of odds ratio and sigma: genes where only their mean differs between classes (only linear), genes where only the correlation structure differs (only nonlinear), and genes where both differ. The title of each subfigure indicates the sampling strategy (binary or continuous) and the sample size (1k or 10k): **A** Binary sampling, N=1k **B** Continuous sampling, N=1k **C** Binary sampling, N=10k. The left three columns of heatmaps in **A-C** contain the mean top-K recovery while the right three columns contain the standard deviation over n=10 replicates. **D** Standard deviation in test AUPRC (n=10 replicates) across simulated datasets with varied odds ratio (linear signal strength) and σ (nonlinear signal strength). Results are shown for binary and continuous data with N=1k and N=10k samples.

bioRxiv preprint doi: https://doi.org/10.1101/2025.11.13.687845; this version posted November 14, 2025. The copyright holder for this preprint (which was not certified by peer review) is the author/funder, who has granted bioRxiv a license to display the preprint in perpetuity. It is made available under aCC-BY 4.0 International license.

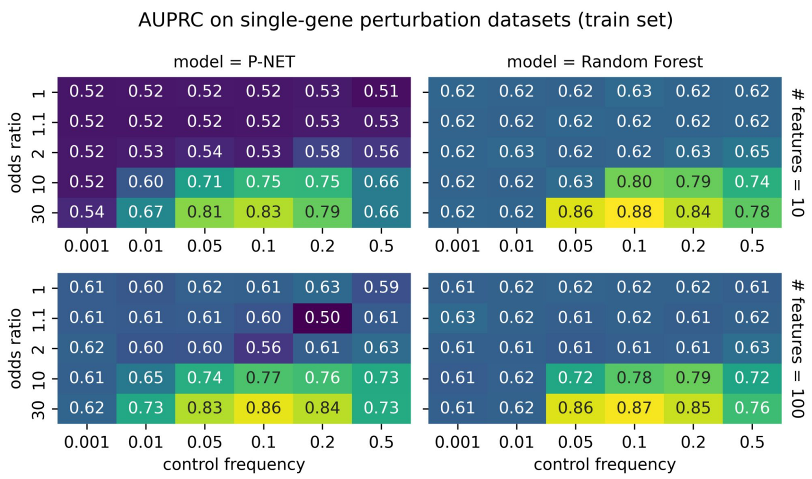

Figure S2. Single-gene perturbation on P1000 somatic mutation backbone evaluated on train set. Heatmaps report the train set AUPRC for each pair of sampling parameters, odds ratio and control frequency. In order, the subpanel columns report results from P-NET and random forest models. The subpanel rows correspond to the number of retained gene features (10 vs. 100, respectively). All values reflect the mean over n=3 replicates.

bioRxiv preprint doi: https://doi.org/10.1101/2025.11.13.687845; this version posted November 14, 2025. The copyright holder for this preprint (which was not certified by peer review) is the author/funder, who has granted bioRxiv a license to display the preprint in perpetuity. It is made available under aCC-BY 4.0 International license.

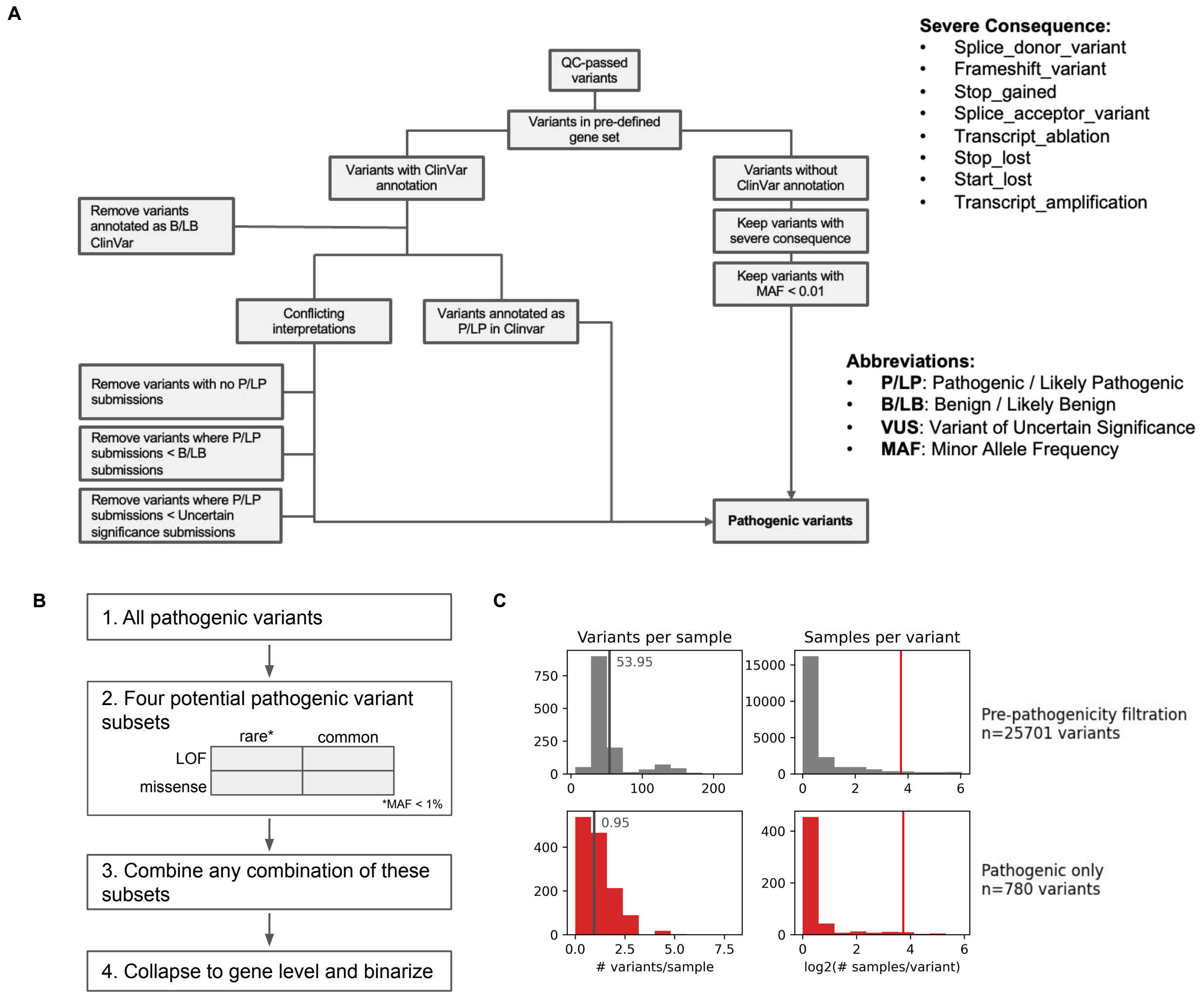

Figure S3. Details on curation of germline datasets. A Flowchart describing process for filtering a VEP-annotated germline VCF to just pathogenic variants. **B** Process for generating various gene-level pathogenic germline datasets (e.g., restricted to rare LOF mutations, or common LOF + common missense mutations). Rare is defined as a minor allele frequency (MAF) < 0.01 in gnomAD. **C** Histograms of variants per sample and samples per variant before and after pathogenicity filtering (first and second columns, respectively). The vertical grey line marks the average number of variants per sample. The vertical red line marks the number of samples corresponding to a dataset-level MAF=0.01.

bioRxiv preprint doi: https://doi.org/10.1101/2025.11.13.687845; this version posted November 14, 2025. The copyright holder for this preprint (which was not certified by peer review) is the author/funder, who has granted bioRxiv a license to display the preprint in perpetuity. It is made available under aCC-BY 4.0 International license.

Top-ranked features (gene x modality) with freq and OR information

Data: somatic only

rank	gene	modality	control_freq	odds_ratio
1	AR	somatic_amp	0.005	130.55
2	PTEN	somatic_del	0.061	5.28
3	TP53	somatic_mut	0.109	4.32
4	AR	somatic_mut	0.005	34.29
5	OBSCN	somatic_del	0.132	0.07
6	AP2A2	somatic_del	0.124	0.12
7	MED30	somatic_amp	0.018	4.55
8	RBBP5	somatic_amp	0.006	14.49
9	COL1A2	somatic_amp	0.118	0.29
10	RHOU	somatic_del	0.092	0.17

Data: germline rare common LOF missense

rank	gene	modality	control_freq	odds_ratio
1	BRCA2	germline_rare_common_lof_missense	0.002	35.2
2	ATM	germline_rare_common_lof_missense	0.012	1.97
3	HLA-A	germline_rare_common_lof_missense	0.008	3.64
4	CNBD1	germline_rare_common_lof_missense	0.009	1.49
5	MSH2	germline_rare_common_lof_missense	0.003	0.0
6	SBDS	germline_rare_common_lof_missense	0.006	3.39
7	ERCC2	germline_rare_common_lof_missense	0.005	1.49
8	ECT2L	germline_rare_common_lof_missense	0.035	0.87
9	TET2	germline_rare_common_lof_missense	0.002	6.75
10	APC	germline_rare_common_lof_missense	0.005	2.24

Data: somatic + germline rare common LOF missense

rank	gene	modality	control_freq	odds_ratio
1	AR	somatic_amp	0.005	130.55
2	PTEN	somatic_del	0.061	5.28
3	TP53	somatic_mut	0.109	4.32
4	AR	somatic_mut	0.005	34.29
5	AP2A2	somatic_del	0.124	0.12
6	OBSCN	somatic_del	0.132	0.07
7	COL1A2	somatic_amp	0.118	0.29
8	MAML3	somatic_amp	0.015	2.99
9	MED30	somatic_amp	0.018	4.55
10	ARF1	somatic_del	0.038	0.17

Figure S4. P-NET's top-ranked features (gene x modality) with the corresponding odds ratio (OR) and control frequencies as calculated from the input datasets. Results reported for three different sets of input data: somatic only, all pathogenic germline LOF and missense variants (rare and common), and their combination.
